# Multiparametric MRI and Machine Learning Based Radiomic Models for Preoperative Prediction of Multiple Biological Characteristics in Prostate Cancer

**DOI:** 10.3389/fonc.2022.839621

**Published:** 2022-02-07

**Authors:** Xuhui Fan, Ni Xie, Jingwen Chen, Tiewen Li, Rong Cao, Hongwei Yu, Meijuan He, Zilin Wang, Yihui Wang, Hao Liu, Han Wang, Xiaorui Yin

**Affiliations:** ^1^ Department of Radiology, Shanghai General Hospital, Shanghai Jiao Tong University School of Medicine, Shanghai, China; ^2^ Institution for Clinical Research, Shanghai General Hospital, Shanghai Jiao Tong University School of Medicine, Shanghai, China; ^3^ Department of Urology, Shanghai General Hospital, Shanghai Jiao Tong University School of Medicine, Shanghai, China; ^4^ Department of Research and Development, Yizhun Medical AI Technology Co. Ltd., Beijing, China; ^5^ Department of Radiology, Jiading Branch of Shanghai General Hospital, Shanghai, China

**Keywords:** radiomics, prostate cancer, magnetic resonance imaging, biological characteristics, risk stratification

## Abstract

**Objectives:**

This study aims to develop and evaluate multiparametric MRI (MP-MRI)-based radiomic models as a noninvasive diagnostic method to predict several biological characteristics of prostate cancer.

**Methods:**

A total of 252 patients were retrospectively included who underwent radical prostatectomy and MP-MRI examinations. The prediction characteristics of this study were as follows: Ki67, S100, extracapsular extension (ECE), perineural invasion (PNI), and surgical margin (SM). Patients were divided into training cohorts and validation cohorts in the ratio of 4:1 for each group. After lesion segmentation manually, radiomic features were extracted from MP-MRI images and some clinical factors were also included. Max relevance min redundancy (mRMR) and recursive feature elimination (RFE) based on random forest (RF) were adopted to select features. Six classifiers were included (SVM, KNN, RF, decision tree, logistic regression, XGBOOST) to find the best diagnostic performance among them. The diagnostic efficiency of the construction models was evaluated by ROC curves and quantified by AUC.

**Results:**

RF performed best among the six classifiers for the four groups according to AUC values (Ki67 = 0.87, S100 = 0.80, ECE = 0.85, PNI = 0.82). The performance of SVM was relatively the best for SM (AUC = 0.77). The number and importance of DCE features ranked first in the models of each group. The combined models of MP-MRI and clinical characteristics showed no significant difference compared with MP-MRI models according to Delong’s tests.

**Conclusions:**

Radiomics models based on MP-MRI have the potential to predict biological characteristics and are expected to be a noninvasive method to evaluate the risk stratification of prostate cancer.

## Introduction

Prostate cancer (PCa) is the highest incidence cancer and the second leading cause of death among men according to the latest statistics in 2021 (1). Early and precise detection of prostate cancer and subsequent appropriate treatment decisions play an essential role for patients (2, 3).

Nowadays, some tumor biomarkers and biological characteristics have been proved to be useful for evaluating the malignant potential of prostate cancer and may influence the treatment decision-making (4–6). Ki67 as a biomarker of cell proliferation, which is expressed in all phases except resting (G0) phase of the cell cycle, has been demonstrated to be an independent prognostic factor in low volume and grade prostate cancer (7). According to the results of the Mayo model, when the expression of Ki67 increased by 1%, the cancer-specific mortality would increase by1 2% after radical prostatectomy (RP) accordingly (8). S100 is a family of acidic calcium-binding proteins and was found to be upregulated in various tumors (9). Aberg et al. revealed two subtypes of S100 were significantly correlated with short progression-free survival in prostate cancer patients with metastases (10). Extracapsular extension (ECE) could be used as an indication of local advanced prostate cancer (cT3). The positive ECE would increase the risk of death to 5 times than the negative ECE for patients after undergoing radical prostatectomy (11). Surgical margin (SM) is determined by pathological staining of RP specimens. Numerous studies have disclosed that positive SM increased cancer-specific mortality and the likelihood of biochemical recurrence of patients significantly (12–14). Prostate cancer tends to invade and grow along nerves, and it is also considered to be a potential metastatic route, which is called perineural invasion (PNI) (15, 16). PNI has been documented to be associated with biochemical recurrence (BCR) and promoting tumor aggressiveness (16, 17). Therefore, judging these biological characteristics before operation can better evaluate the invasiveness of tumor and may change the clinical decision-making patterns in the future.

Definitely, biopsy can solve some of the above problems to a certain extent and is still the mainstream method. It is reported that the combined technique of fusion targeted and systematic biology has been proved to be helpful to improve the diagnostic accuracy (18). However, the defects of sampling errors and a series of subsequent complications, such as pain and hematuria (19), limit the real-time monitoring and accurate evaluation of biological characteristics by biopsy. Multiparametric-magnetic resonance imaging (MP-MRI) is one of the most accurate noninvasive methods to evaluate local lesions, which contains T1 and T2 sequences that provide anatomical and disease information, as well as other sequences that provide functional information, such as diffusion-weighted imaging (DWI), dynamic contrast enhanced (DCE), and magnetic resonance spectroscopy (MRS) (20). As a routine screening method for prostate cancer, MP-MRI can reflect the phenotype and heterogeneity of prostate cancer by signal intensity and enhancement features (21, 22). Furthermore, MP-MRI images may contain many clinically valuable information related to the different biological characteristics above, such as ECE (23), which may be hard for radiologists to dig out in clinical practice.

Currently, radiomics serves as a novel approach that extracts abundant quantitative features with high throughput, and through machine learning methods to establish prediction models, which were proved to effectively provide more potential useful information for the clinical practice in urology (24, 25). Radiomics of prostate cancer has been widely used in tumor identification, staging, and prognosis evaluation (26, 27). However, more comprehensive and accurate prediction models that can determine the risk stratification and provide references for clinical decision-making for prostate cancer still need to be explored.

Thus, in the present study, we attempted to establish and validate the radiomic predictive models for five biological characteristics related to aggressiveness (Ki67, S100, ECE, PNI, SM) of prostate cancer based on MP-MRI. In addition, some clinical information was also added to establish the corresponding combined models.

## Materials and Methods

### Patients, Pathological Evaluation, and MRI Acquisition

This work was approved by the Institutional Review Board (IRB) of Shanghai General Hospital (2021KY107), and the patient’s informed consent was authorized to be waived according to the nature of the research. This retrospective study collected patients who underwent radical prostatectomy from May 2013 to January 2020. The exclusion criteria are as follows: (1) Preoperative DCE-MRI were unavailable (*n* = 149); (2) No mass lesion found on MRI image (*n* = 9); (3) Missing DWI (*n* = 3); (4) Poor imaging quality (*n* = 10); (5) Missing clinical information (*n* = 5); (6) Biopsy before MRI leading to unclear lesions (*n* = 32); and (7) Previous treatment before MRI examinations (*n* = 12). Finally, we recruited 252 patients as our subjects.

The clinical and pathological information we collected in this study is as follows: age, prostate serum antigen (PSA), white blood cell (WBC), red blood cell (RBC), hemoglobin, lymphocyte, platelet, albumin, alkaline phosphatase (ALP), platelet-to-lymphocyte ratio (PLR), fibrinogen, surgical Gleason score, immunohistochemistry (Ki67, S100, AR), SM, ECE, PNI, seminal vesicle invasion (SVI), and lymphatic vascular invasion (LVI). Details of the above indicators can be found in **Supplementary Table S2**. Considering the importance of biological characteristics of prostate cancer mentioned above, and the routine indexes of pathological examination, as well as data distribution (balanced or imbalanced), we selected five of them (Ki67, S100, ECE, PNI, SM) as our research indicators. Each of these indicators was classified as a group, and we divided each group into training cohort and validation cohort in the ratio of 4:1. All indicators were divided into positive and negative in the form of binary classification, except Ki67, which was divided into high expression and low expression with 10% as the threshold according to previous studies (7). The gold standard references of this research were based on the results of radical prostatectomy.

Three MRI sequences were included in this study: T2, DWI, DCE (arterial phase), and this combination also meets the PI-RADS v2 (Prostate Imaging-Reporting and Data System, Version 2) standard of MP-MRI (28). The protocols of MRI examinations are described in **Supplement A**.

### Tumor Segmentation

**Figure 1:**
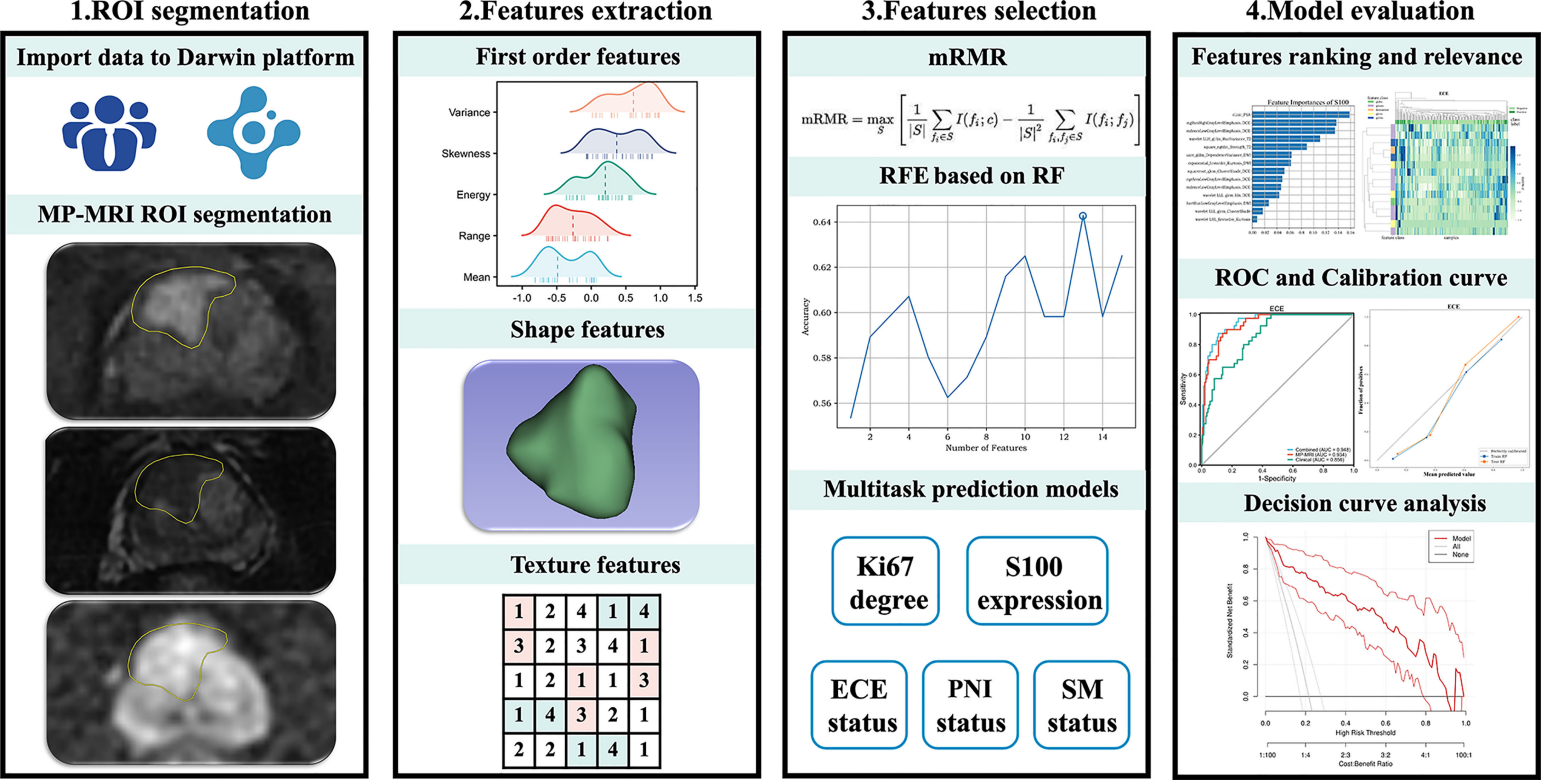
The general workflow of this study.

The patient’s images and clinical data were imported into the Darwin research platform (https://arxiv.org/abs/2009.00908) for subsequent tumor lesion delineation and model establishment. The work flow is shown in **Figure 1**. The boundary of the volume of interest (VOIs) on each axial-DWI picture was manually delineated by radiologist 1 (JC, 5 years of experience in urinary imaging). The ROIs on DWI were then copied to the sequences of T2 and DCE. If some of the copied results of the two sequences were not ideal, further modifications were made. Next, radiologist 2 (RC, 8 years of experience in urinary imaging) would review the segmentation results. If there was any objection to the results, the results would be discussed and resegmented until a consensus was reached.

### Feature Extraction

After finishing segmentation, the feature extraction of lesions was carried out by PyRadiomics package. The original feature classes contain first-order, shape, and texture features. First-order features refer to the distribution of voxel intensities through general and basic metrics, such as range, mean, variance, and kurtosis. Texture features embody: Gray-Level Cooccurrence Matrix (GLCM); Gray-Level Run Length Matrix (GLRLM); Gray-Level Size Zone Matrix (GLSZM); Neighboring Gray-Tone Difference Matrix (NGTDM); and Gray-Level Dependence Matrix (GLDM). Furthermore, we use eight filters and the original images were derived into eight kinds of filtered transformed images: Laplacian of Gaussian (LoG), wavelet, square, square root, logarithm, exponential, gradient, and local binary pattern (LBP). Except shape features, the first-order and texture features mentioned above can also be extracted from the derived images. Due to a single MRI sequence containing 1,781 features, MP-MRI produced 5,343 features in total for this study. The detailed description of the image features mentioned above can be found in https://pyradiomics.readthedocs.io/en/latest/features.html.

### Feature Selection

Firstly, the extracted feature data were subtracted by the mean and then divided by the variance to achieve data normalization for subsequent comparison. Next, in order to reduce the over fitting of data and find the optimal correlation features, max relevance min redundancy (mRMR) was adopted to find the top 20 features for ECE, PNI, and SM groups and 15 features for Ki67 and S100 groups in the training cohort. Due to some machine learning, classifiers themselves can evaluate the importance of features and find the best feature combination through multiple iterative calculations. Therefore, the recursive feature elimination (RFE) based on random forest (RF) was applied to find the best feature combination step by step based on accuracy.

### Model Construction

In this study, six classifiers were included to find the best diagnostic performance among them: support vector machine (SVM), K-nearest neighbor (KNN), random forest, decision tree, logistic regression, and XGBOOST. Support vector machine was based on polynomial kernel function, and the tolerance for misclassified samples was set by the specific penalty coefficient C (from 0.0001 to 1,000). The best *k* value (number of neighbors) for KNN was found by training in the range of 3–10. For random forest, decision tree, and XGBOOST, the maximum tree depth was constrained to avoid overfitting. To find out whether the clinical data improved the diagnostic performance, several clinical data (age, PSA, WBC, RBC, hemoglobin, lymphocyte, platelet, albumin, ALP, PLR, fibrinogen) were selected to build the clinical models. Meanwhile, they were integrated into the corresponding MP-MRI radiomic models to construct the combined models. The parameters used in the model construction are described in **Supplementary Table S1**.

### Model Evaluation and Statistical Analysis

The diagnostic efficiency of the models was demonstrated by receiver operating characteristic (ROC) curves and quantified by the area under the curve (AUC). The calibration curve shows the consistency between the prediction model and the actuality. What is more, the decision curve analysis (DCA) illustrated the clinical net benefits brought by the prediction model.

The diagnostic ability of MP-MRI models and combined models was compared by DeLong’s test. The overall comparison of PSA in each group was through Mann-Whitney *U* test. The case distribution between validation cohorts and training cohorts was compared by Chi-square test. All statistical analysis was performed by R (version 4.0.2). The statistically significant level was set at 0.05.

## Results

### Demographics

In this study, a total of 252 PCa patients were included, and the flowchart of patients’ recruitment is depicted in **Supplementary Figure S1**. The baseline characteristics of PCa patients could be found in **Supplementary Table S2**. The mean age of the included patients was 68.4 years (50–84 years), and their surgical Gleason score is mainly distributed in 7 (64.7%). According to their pathological results, the included patients were divided into 5 groups: Ki67 (*n* = 140), S100 (*n* = 158), ECE (*n* = 232), PNI (*n* = 225), and SM (*n* = 248). As shown in **Table 1**, the expression of PSA was significantly different in ECE (*p* < 0.01), PNI (*p* = 0.03), and SM (*p* < 0.01) groups, and it was relatively not significant in Ki67 (*p* = 0.08) and S100 (*p* = 0.12) groups. Meanwhile, the case composition between the cohort training and validation cohort was roughly the same in each group (*p* > 0.05).

**Table 1:** Patient profiles of each group.

Characteristic	PSA (ng/ml)	Training cohort	Validation cohort
***Ki67***		*n* = 112	*n* = 28
≥10%	19.0 ± 15.4	38 (33.9%)	9 (32.1%)
<10%	15.6 ± 15.4	74 (66.1%)	19 (67.9%)
*p*-value	0.08	0.86	
***S100*, *n* (%)**		*n* = 126	*n* = 32
Positive	16.0 ± 11.9	67 (53.2%)	17 (53.1%)
Negative	14.7 ± 14.4	59 (46.8%)	15 (46.9%)
*p*-value	0.12	1.00	
**ECE, *n* (%)**		*n* = 185	*n* = 47
Positive	25.2 ± 22.5	40 (21.6%)	10 (21.3%)
Negative	13.9 ± 14.5	145 (78.4%)	37 (78.7%)
*p*-value	<0.01	0.96	
**PNI, *n* (%)**		*n* = 180	*n* = 45
Positive	18.3 ± 18.9	96 (53.3%)	24 (53.3%)
Negative	13.9 ± 14.3	84 (46.7%)	21 (46.7%)
*p*-value	0.03	1.00	
**SM, *n* (%)**		*n* = 198	*n* = 50
Positive	22.7 ± 22.8	129 (65.2%)	32 (64%)
Negative	12.4 ± 10.6	69 (34.8%)	18 (36%)
*p*-value	<0.01	0.88	

The comparison of PSA in each group was by Mann-Whitney U test. The case distribution between validation cohorts and training cohorts was compared by Chi-square test. ECE, extracapsular extension; PNI, perineural invasion; SM, surgical margins.

### Feature Selection

**Figure 2:**
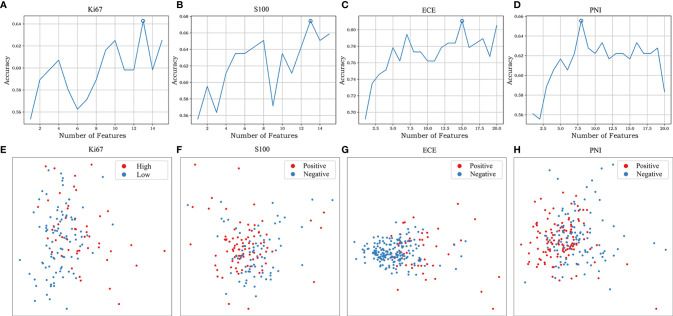
The feature selection of RFE-RF and the distribution of different cases on PCA. RFE-RF **(A–D)** was applied to find the best feature combination step by step, and the combinations with the highest accuracy will be incorporated into the models. PCA **(E–H)** showed the selected features could satisfactorily distinguish the division of cases in each group intuitively according to their feature values. The corresponding figures for SM are shown in **Supplementary Figure S2**.

**Figure 3:**
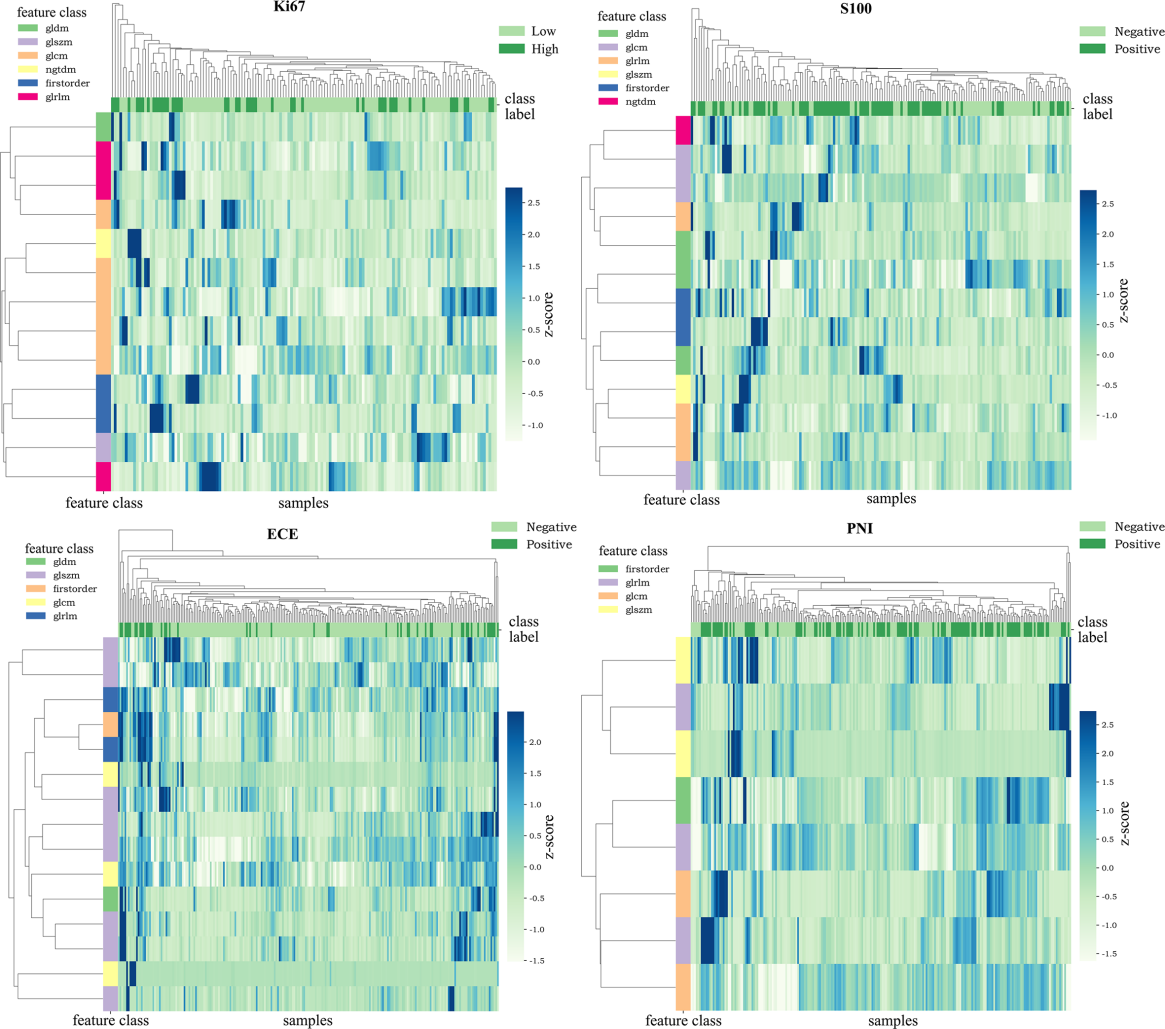
Heat maps of the selected features. The color of the maps represented the value of the selected features. The color of positive cases or high Ki67 expression cases was generally darker. This proved the ability of the features themselves to distinguish the biological characteristics of patients.

After applying mRMR to the features extracted from MP-MRI, the top 20 features in ECE, PNI, and SM groups and 15 features in Ki67 and S100 groups were obtained in the training cohort. RFE-RF then selected the resulting features and achieved the best performing feature combination as shown in **Figures 2A–D**: Ki67 (*n* = 13), S100 (*n* = 13), ECE (*n* = 15), PNI (*n* = 8), and SM (*n* = 20). For SM, all its figures are placed in **Supplementary Figure S2** for a better result demonstration. Next, principal component analysis (PCA) was performed to extract principal components of features in each group and reduce the dimensions, which made the division of cases in each group more intuitive according to their feature values. As displayed in **Figures 2E–H**, the selected features could satisfactorily distinguish the positive and negative cases on PCA (for Ki67, they were ≥10% and <10%), especially in the ECE and PNI groups, which successfully divided the cases with different labels into left sides and right sides. According to the heat maps in **Figure 3**, the color of positive cases or high Ki67 expression cases was generally darker, and the color in heatmaps referred to the values of the selected feature. This also proved the ability of the features themselves to distinguish the biological characteristics of patients. **Supplementary Figure S3** showed that the correlation among radiomics features was weak, indicating there was low redundancy among selected features.

### Comparison of Different Classifiers

The six classifiers (SVM, KNN, random forest, decision tree, logistic regression, and XGBOOST) and their AUC in each group are listed in **Table 2**. In general, the performance of random forest was the best according to AUC values, and we chose random forest as the prediction models for the four groups (Ki67 = 0.87, S100 = 0.80, ECE = 0.85, PNI = 0.82). As for SM, the performance of SVM was relatively the best (AUC = 0.77), so SVM with polynomial kernel function was selected as the optimal classifier for SM.

**Table 2:** Diagnostic performance of optimal models for each group.

Different models	Training cohort					Validation cohort				
	AUC	SEN	SPE	ACC	*p-*value	AUC	SEN	SPE	ACC	*p-*value
***Ki67***										
MP-MRI	0.91	0.92	0.76	0.81	0.59	0.87	1.00	0.58	0.71	0.60
Clinical	0.73	0.53	0.84	0.73		0.63	0.67	0.74	0.71	
Combined	0.91	0.92	0.76	0.81		0.88	0.78	0.84	0.82	
***S100***										
MP-MRI	0.88	0.81	0.81	0.81	<0.01	0.80	0.82	0.71	0.75	0.58
Clinical	0.85	0.82	0.69	0.76		0.66	0.62	0.53	0.63	
Combined	0.94	0.84	0.92	0.87		0.81	0.94	0.60	0.78	
**ECE**										
MP-MRI	0.93	0.88	0.86	0.86	0.01	0.85	1.00	0.62	0.70	0.91
Clinical	0.86	0.98	0.57	0.65		0.57	0.50	0.84	0.77	
Combined	0.95	0.88	0.88	0.88		0.85	0.80	0.73	0.74	
**PNI**										
MP-MRI	0.87	0.84	0.79	0.82	<0.01	0.82	0.67	0.95	0.80	0.19
Clinical	0.81	0.88	0.68	0.78		0.58	0.67	0.52	0.60	
Combined	0.89	0.85	0.80	0.83		0.84	0.71	0.90	0.80	
**SM**										
MP-MRI	0.87	0.83	0.78	0.80	0.01	0.77	0.72	0.72	0.72	0.97
Clinical	0.84	0.83	0.74	0.77		0.65	0.71	0.47	0.64	
Combined	0.94	0.96	0.81	0.86		0.77	0.61	0.81	0.74	

The p-values were derived from DeLong’s test, and they compare the AUCs of the MP-MRI models with the corresponding combined model. The models of SM were based on SVM; the others were based on RF.

### Performance of MP-MRI and Combined Prediction Models

**Figure 4:**
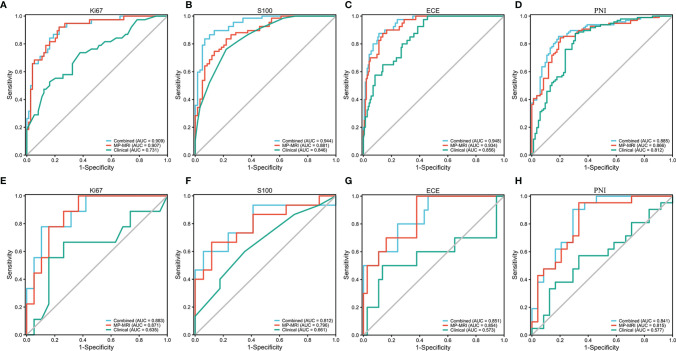
The ROC curves of the MP-MRI, clinical and combined models in the training cohort **(A–D)** and validation cohort **(E–H)**.

The optimal MP-MRI models of the five groups performed satisfactorily both in the training cohorts and validation cohorts (**Figure 4**; **Table 3**). The prediction model of Ki67 performed best among the five groups, whose AUC value reached 0.88 in the validation cohort. The second-best model was ECE with AUC value = 0.85. The AUC values of the three remaining models in the validation cohort were 0.80 for S100, 0.82 for PNI, and 0.77 for SM.

**Table 3:** AUCs of different MP-MRI radiomic classifiers for predicting the five biological characteristics in the validation cohorts.

Classifiers	*Ki67*	*S100*	ECE	PNI	SM
**Random forest**	**0.87**	**0.80**	**0.85**	**0.82**	0.72
**Decision tree**	0.75	0.76	0.77	0.72	0.75
**SVM**	0.84	0.79	0.84	0.78	**0.77**
**KNN**	0.74	0.70	0.82	0.72	0.72
**Logistic regression**	0.75	0.80	0.82	0.81	0.68
**XGBOOST**	0.76	0.70	0.74	0.73	0.67

The bold values represent the AUC of the classifiers that perform best in each subgroup.

SVM, support vector machine; KNN, K-nearest neighbor.

As for clinical factors, after REF-RF selection, 2 characteristics were included for Ki67 (PSA, PLR), 1 for S100 (PSA), 4 for ECE (PSA, WBC, PLR, and ALP), 5 for PNI (PLR, age, fibrinogen, PSA, and albumin), and 4 for SM (PSA, fibrinogen, albumin, and lymphocyte). This displayed the level of PSA might be helpful to distinguish five biological characteristics to some extent. Clinical characteristics were then added to the MP-MRI model to form the combined models. As a result, in the training cohort, the combined model was significantly better than the MP-MRI models except the Ki67 group based on Delong’s tests (*p* < 0.05). Nevertheless, in the validation cohort, there was no significant difference between the two groups (*p* > 0.05).

**Figure 5:**
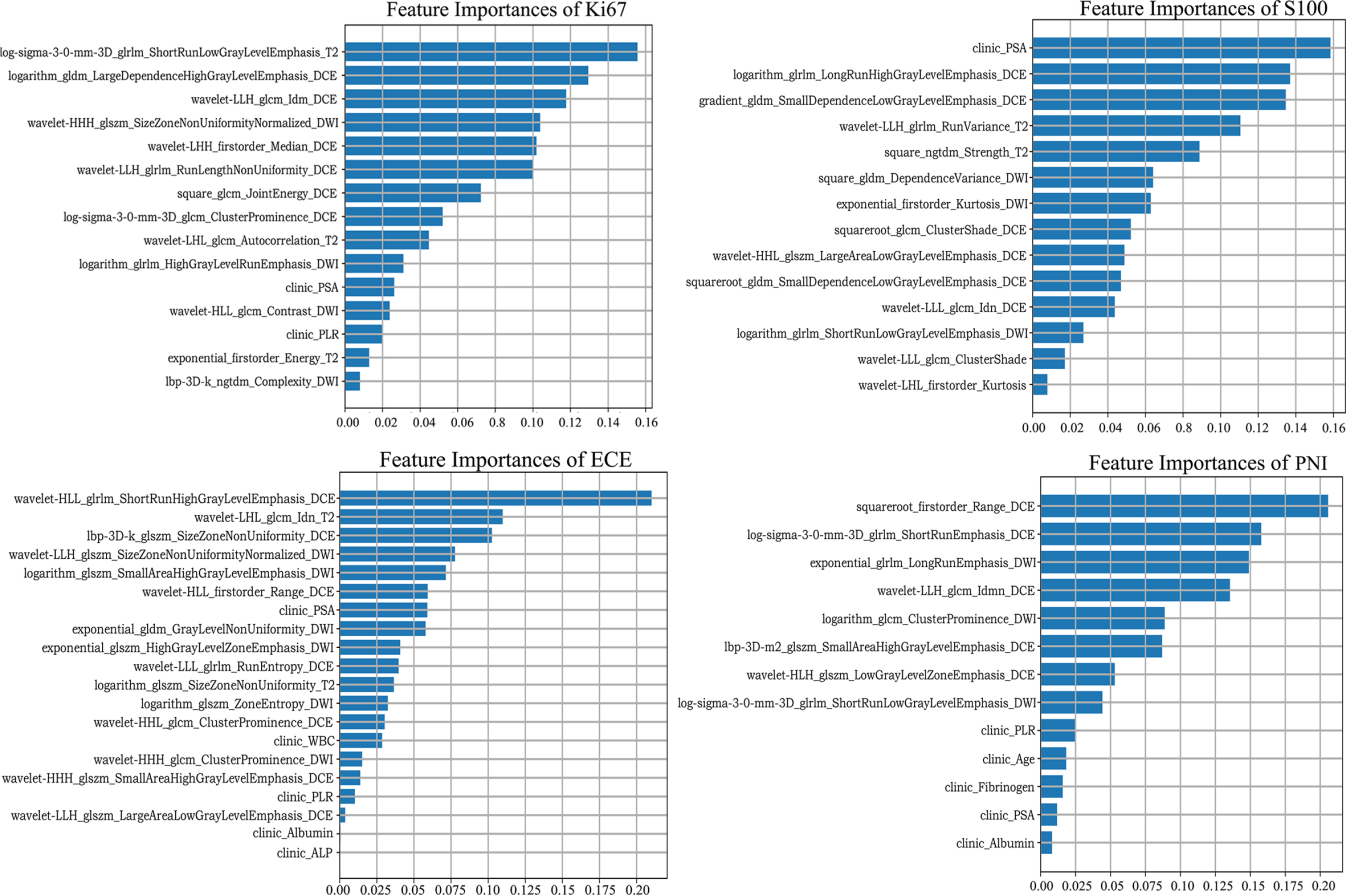
The inbuilt feature importance in each combined model.

**Figure 6:**
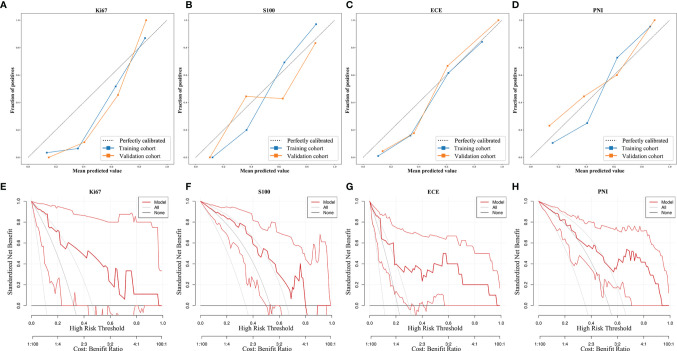
The calibration curves and decision curve analysis of the MP-MRI models. The calibration curves **(A–D)** show the consistency between the prediction model and the actuality. The dotted reference line indicated perfect calibration. The DCA **(E–H)** illustrated the clinical net benefits brought by the prediction model. The gray line indicated “treat all,” and the black horizontal line indicated “treat none”.

**Figure 7:**
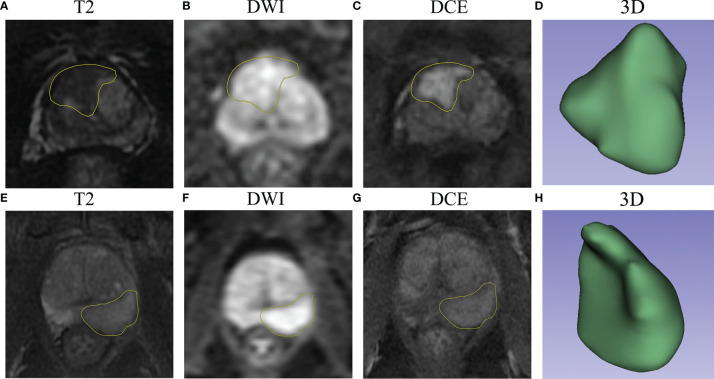
The examples of VOI delineation on MP-MRI. **(A–D)** A 66-year-old patient was pathologically diagnosed as PNI positive with a typical abnormal signal lesion in the right front of the prostate. **(E–H)** A 70-year-old patient was diagnosed as PNI negative with a lesion located in the left peripheral zone, and the DCE sequence showed moderate enhancement. **(D, H)** represent the 3-dimensional reconstruction of the VOI.

Furthermore, the importance of the features in the combined models is demonstrated in **Figure 5**. The number and importance of DCE features ranked first in models of each group, followed by DWI, and finally T2. This also revealed that DCE sequences could provide more information for predicting the malignant degree of prostate cancer. In addition, in **Figure 6**, calibration curves displayed the consistency between the prediction model and the actuality was favorable, and when the risk threshold is greater than about 0.1, the prediction model could bring more clinical net benefits according to the DCA. Finally, we provide the examples of VOI delineation on MP-MRI and the corresponding 3D constructions images in **Figure 7**.

## Discussion

In this study, we constructed the machine-learned radiomic models based on six classifiers for the five biological characteristics (Ki67, S100, ECE, PNI, and SM) related to the invasiveness of prostate cancer. ROC curves showed that the diagnostic abilities of these models were ideal with AUC values all greater than 0.8 in the validation cohorts (except SM = 0.77). Meanwhile, we added several clinical characteristics to make the combined models, though they barely improved the accuracy of prediction.

For traditional diagnostic methods, like serum PSA test, digital rectal examinations (DREs), and prostate biopsy, they inevitably have many major deficiencies (19, 29–31). The main deficiencies are that they may lead to overdiagnosis of prostate cancer and missed diagnosis of clinically significant cancer (32–34). As shown in **Supplementary Table S2**, 22.6% of patients showed Gleason score 6 or less, suggesting that a relevant number of cancers below the threshold which is currently considered clinically significant cancer and leading to overdiagnosis and overtreatment. MP-MRI as a noninvasive method has been recommended as a routine examination of prostate cancer and proved to be beneficial in the detection of clinically significant cancer (21, 22). Recently, the NCCN guidelines clearly pointed out that MP-MRI was helpful to the staging and risk stratification of prostate cancer, and its combination with several biomarkers could reduce unnecessary biopsy (35). Moreover, MP-MRI contains much clinically valuable information, which has not attracted enough attention in clinical practice. Recently, artificial intelligence, such as radiomics, has shown great potential for evaluating the aggressiveness of urological tumors (36). Therefore, radiomics could be used as a novel and efficient way to dig out the information (24). Radiomics has been applied to predict many aspects of prostate cancer, such as cancer diagnosis, Gleason score, treatment response, and early biochemical recurrence (37).

Nowadays, using radiomics to predict multiple biological characteristics of tumors simultaneously has become a trend. In the research of Meng et al. (38), they proposed the radiomic models based on MP-MRI have the ability to predict multiple biological characteristics (HER-2, Ki67, differentiation, lymph node metastasis, and KRAS-2) of rectal cancers. However, to the best of our knowledge, there were fever researches to comprehensively predict various biological characteristics of prostate cancer using radiomics and achieved good diagnostic results. Bai et al. (39) reported their radiomic model could predict the presence of ECE preoperatively, but the AUC value of their integrated model was only 0.71, much lower than ours (AUC = 0.85). He et al. (40) used MP-MRI radiomics to predict ECE (AUC = 0.728, also lower than ours) and SM (AUC = 0.76, similar to ours), yet they did not comprehensively evaluate the aggressiveness of prostate cancer as ours. Therefore, our comprehensive radiomic models made it possible to predict more critical biological characteristics of prostate cancer and improve the prediction accuracy of some biological characteristics compared with the other published AI models.

In the present study, we extracted as many features as the recent literature documented. We then adopted an efficient feature selection method—mRMR, which has been proved advanced in a majority of researches (38, 41), to obtain the most relevant and least redundant features. In addition, the low redundancy of selected features could be testified by the correlation maps in **Supplementary Figure S3**. RFE-RF then ensured the best combinations of the included features. More and more studies use RFE-RF to select the best feature combinations, yet it needs a large amount of computation so that it is suitable for low-dimensional data after primary selection (42).

For the resulting radiomic features of each group, wavelet features account for larger proportions: 6/13 for Ki67, 5/13 for S100, 9/16 for ECE, 2/8 for PNI, and 7/20 for SM. Wavelet features are derived from the wavelet transform and represent high-dimensional features that cannot be easily deciphered by humans (43). Wavelet features could show the heterogeneity of tumors, and multiple studies have proved its strong prediction ability (38, 44). In addition, DCE sequences occupied a large part of the models. The reason may be that prostate cancer has strong ability of neovascularization, and the morphology and density of neovascularization are closely related to metastasis and prognosis of patients (45, 46). DCE-MRI is exactly a fairly adequate way to demonstrate neovascularization. Also, because of the increased vascular permeability of prostate cancer, we chose the arterial phases of DCE sequences to delineate the lesions (28).

Classifiers play an essential role in machine learning. The six classifiers that were included in this study: SVM, KNN, random forest, decision tree, logistic regression, and XGBOOST. As a result, the classification performance of random forest was generally the best among them. Random forest is composed of a large number of decision trees, and its prediction result is averaged by all the tree predictions, so it can effectively avoid over fitting. It has been documented that random forest occupied a large part of the Kaggle Data Science Competitions and ranked first among 179 classifiers (47).

It is inevitable that there are some coexisting prostatic diseases in patients with prostate cancer. In our study, coexisting diseases contained benign prostatic hyperplasia, chronic prostatitis, prostatic cyst, etc. However, they did not have a great impact on our study and our models still achieved favorable distinguishing ability. The reason may be that the features selected by our screening methods have strong specificity for the corresponding biological characteristics, and many of them reflect the complexity of the lesions, such as texture features (48). For coexisting diseases like prostatic hyperplasia, the density of lesions was relatively more consistent on MP-MRI and they would not make a remarkable difference to the accuracy of our models. The established model should be more applicable to clinical reality. If they were only applied to target diseases and excluding coexisting diseases, the clinical application of the models would be seriously limited.

Although radiomics shows huge potential for the improvement of clinical diagnosis and risk stratification, its practical clinical application is still subject to many difficulties, and its real benefits are required to be further confirmed in prospective cohort studies (49). However, radiomics plays an increasingly important role in medical imaging, and it provides a unique basis for personalized precision treatment (50). In our study, we proved the applicability of radiomics in predicting the multiple biological characteristics of prostate cancer, and we also provided relatively detailed protocol for MP-MRI and key machine-learning parameters to offer a reference for the standardization work in the future (51). The next main steps of radiomics could be to take advantage of deep learning methods (for example, U-Net) to delineate the ROI automatically and to prove the robustness of the radiomic models through multicenter, prospective, randomized-controlled trials (52).

This study had the following limitations: Firstly, this study was a retrospective and single-center study, and this inevitably led to selection bias and lack of samples and external verification. Secondly, some valuable biological characteristics or biomarkers were not included in the model due to incomplete data, for example, gene mutation data, which had great guiding significance for clinical treatment. Thirdly, our models were not as intuitive as a nomogram due to the algorithm of random forest and SVM with polynomial kernel function. Fourthly, the delineation of lesions was performed manually instead of computer-aided, which may lead to inconsistencies in clinical practice. Therefore, our next research focus will be put on multicenter, prospective, more clinically feasible, large-scale, and valuable indicator-based studies.

## Conclusion

The present work associated the radiomics features of MP-MRI with five biological characteristics related to the aggressiveness of prostate cancer. The established comprehensive models made it possible to predict more critical biological characteristics of prostate cancer and achieved favorable prediction abilities. Therefore, the models are expected to noninvasively evaluate the risk stratification of prostate cancer and provide valuable guidance for clinical decision-making.

## Data Availability Statement

The raw data supporting the conclusions of this article will be made available by the authors, without undue reservation.

## Ethics Statement

The studies involving human participants were reviewed and approved by the Institutional Review Board (IRB) of Shanghai General Hospital (2021KY107). The ethics committee waived the requirement of written informed consent for participation. Written informed consent was not obtained from the individual(s) for the publication of any potentially identifiable images or data included in this article.

## Author Contributions

XY, HW, and XF designed the study. NX, TL, RC, and HY acquired the data. MH, ZW, and HL analyzed the data. XF and NX wrote the report, which was edited by all authors. YW and XY supervised the project. All authors contributed to the article and approved the submitted version.

## Funding

This research is financially supported by the National Natural Science Foundation of China (81871400), the Project of Shanghai Science and Technology Committee (19411951403), and the Key Project of Shanghai Education Commission (202101070002E00085).

## Conflict of Interest

Author HL was employed by Yizhun Medical Technology Co. Ltd., Beijing, China.

The remaining authors declare that the research was conducted in the absence of any commercial or financial relationships that could be construed as a potential conflict of interest.

## Publisher’s Note

All claims expressed in this article are solely those of the authors and do not necessarily represent those of their affiliated organizations, or those of the publisher, the editors and the reviewers. Any product that may be evaluated in this article, or claim that may be made by its manufacturer, is not guaranteed or endorsed by the publisher.
