# Supplementary material for: Multiparametric MRI and Machine Learning Based Radiomic Models for Preoperative Prediction of Multiple Biological Characteristics in Prostate Cancer
Source: Front Oncol. 2022 Feb 7;12:839621. doi: 10.3389/fonc.2022.839621 (PMC8859464; doi:10.3389/fonc.2022.839621)
Supplement: Supplementary file 1 [file Image_1.pdf]

Supplementary Material

Supplementary A: The protocol of prostate MP-MRI

The MRI examinations were performed using the 3.0T MRI scanners (Discovery MR750 3.0T and Discovery MR750w 3.0T, General Electric Medical Systems, Milwaukee, USA; Magnetom Prisma 3.0T, Siemens Healthineers, Erlangen, Germany) with 8-channel phased-array pelvis coils, and the patients were in supine position. The details of the MP-MRI sequences in this study were as follows:

1. The oblique axial T2WI fs sequence: repetition time (TR)/echo time (TE), 4500-4700/102ms, Bandwidth 83.333KHz, Thickness 5.0mm, Spacing 1.0mm, FOV 16cm, Frequency 288, Echo Train Length 28, NEX 3.0, Refocus angle 110°.

2. The oblique axial DWI (b= 1000 or 1500 s/mm2) Shim sequence: TR/TE, 4511/83.6ms, Bandwidth 250.0KHz, Thickness 4.0mm, Spacing 1.0mm, Frequency 128, Phase 128, NEX 6.0.

3. The dynamic contrast-enhanced (DCE) axial scan using a spoiled gradient echo sequence: TR/TE 8.5/1.3&2.3ms, Bandwidth 142.86KHz, Thickness 4.0mm, Frequency 160, Phase 192, NEX 1.0, Flip angle 12°. All patients were injected with 0.1mmol/kg Gd-DTPA at 2.0 ml/s, and four repetitions were acquired: initial, 21s, 44s and 65s after the injection.

Supplementary Table 1. The parameters used in the construction of MP-MRI models

Classifiers	Parameters	Ki67	S100	ECE	PNI	SM
**Random forest**	max_depth	4	4	3	4	5
	min_samples_leaf	17	22	12	20	24
**Decision tree**	max_depth	7	8	6	7	5
	min_samples_leaf	10	12	13	16	17
**SVM**	C	0.5	1	0.07	0.4	0.04
	kernel function	polynomial	RBF	polynomial	polynomial	polynomial
**KNN**	n_neighbors	6	8	8	5	5
**Logistic regression**	l1_ratio	0.5	0.5	0.5	0.5	0.5
**XGBOOST**	max_depth	4	6	3	4	4
	min_child_weight	9	13	10	12	14

RBF, radial basis function

Supplementary Table 2. The baseline characteristics of PCa patients

Characteristic	Values
**Total number**	252
**Age (y), mean (range)**	68.4 (50-84)
**PSA (ng/ml), mean (IQR)**	16.1 (7.0, 19.1)
**WBC ($\times 10^9$/L), mean (SD)**	6.0 (2.2)
**RBC ($\times 10^{12}$/L), mean (SD)**	4.5 (0.5)
**Hemoglobin (g/L), mean (SD)**	138.9 (13.1)
**Lymphocyte ($\times 10^9$/L), mean (SD)**	1.7 (0.8)
**Platelet ($\times 10^9$/L), mean (SD)**	193.2 (51.5)
**Albumin (g/L), mean (SD)**	41.82 (3.7)
**ALP (U/L), mean (SD)**	65.8 (16.1)
**Fibrinogen (g/L), mean (SD)**	2.6 (0.6)
**PLR, mean (SD)**	131.0 (60.9)
**Surgical Gleason score, n (%)**	
≤6	57 (22.6%)
7	163 (64.7%)
8	9 (3.6%)
≥9	23 (9.1%)
**Immunohistochemistry**	
Ki67, n (%)	47 (18.7%)
<10%	93 (36.9%)
≥10%	112 (44.4%)
No date	
S100, n (%)	
Positive	84 (33.3%)

Negative	74 (29.4%)
No date	94 (37.3%)
AR, n (%)	
Positive	174 (69.0%)
Negative	7 (2.8%)
No date	71 (28.2%)
**Surgical margins, n (%)**	
Positive	87 (34.5%)
Negative	161 (63.9%)
No date	4 (1.6%)
**Extracapsular extension, n (%)**	
Positive	50 (19.8%)
Negative	182 (72.2%)
No date	20 (7.9%)
**Perineural Invasion, n (%)**	
Positive	120 (47.6%)
Negative	105 (41.7%)
No date	27 (10.7%)
**Seminal vesicle invasion, n (%)**	
Positive	21 (8.3%)
Negative	225 (89.3%)
No date	6 (2.4)
**Lymphatic vascular invasion, n (%)**	
Positive	19 (7.5%)
Negative	150 (59.5%)
No date	83 (32.9%)

PSA, prostate serum antigen; WBC, white blood cell; RBC, red blood cell; ALP, alkaline phosphatase; PLR, platelet-to-lymphocyte ratio.

Supplementary Figure 1. Flowchart of patients' recruitment.

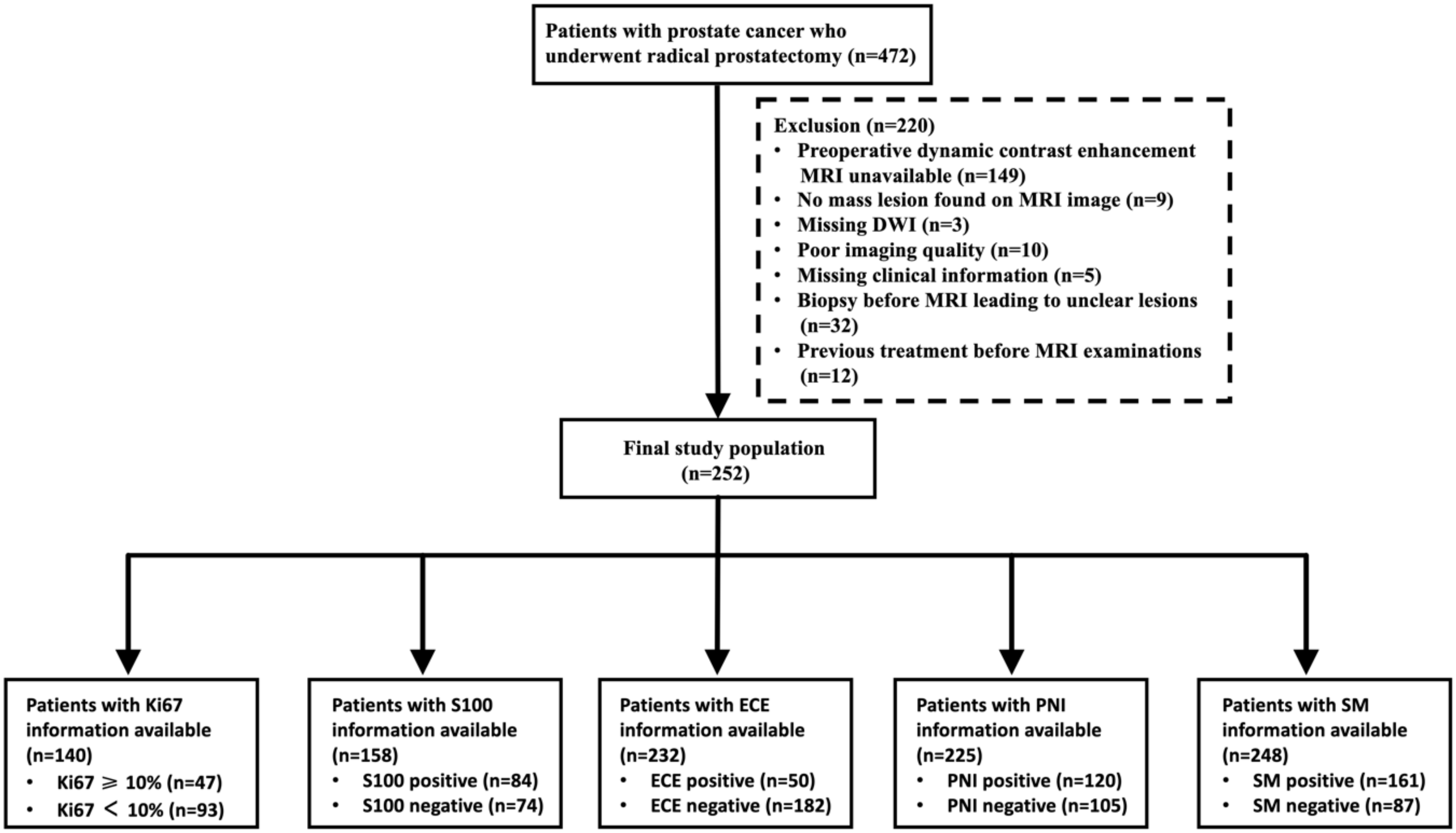

Supplementary Figure 2. The features selection and model evaluation process of SM.

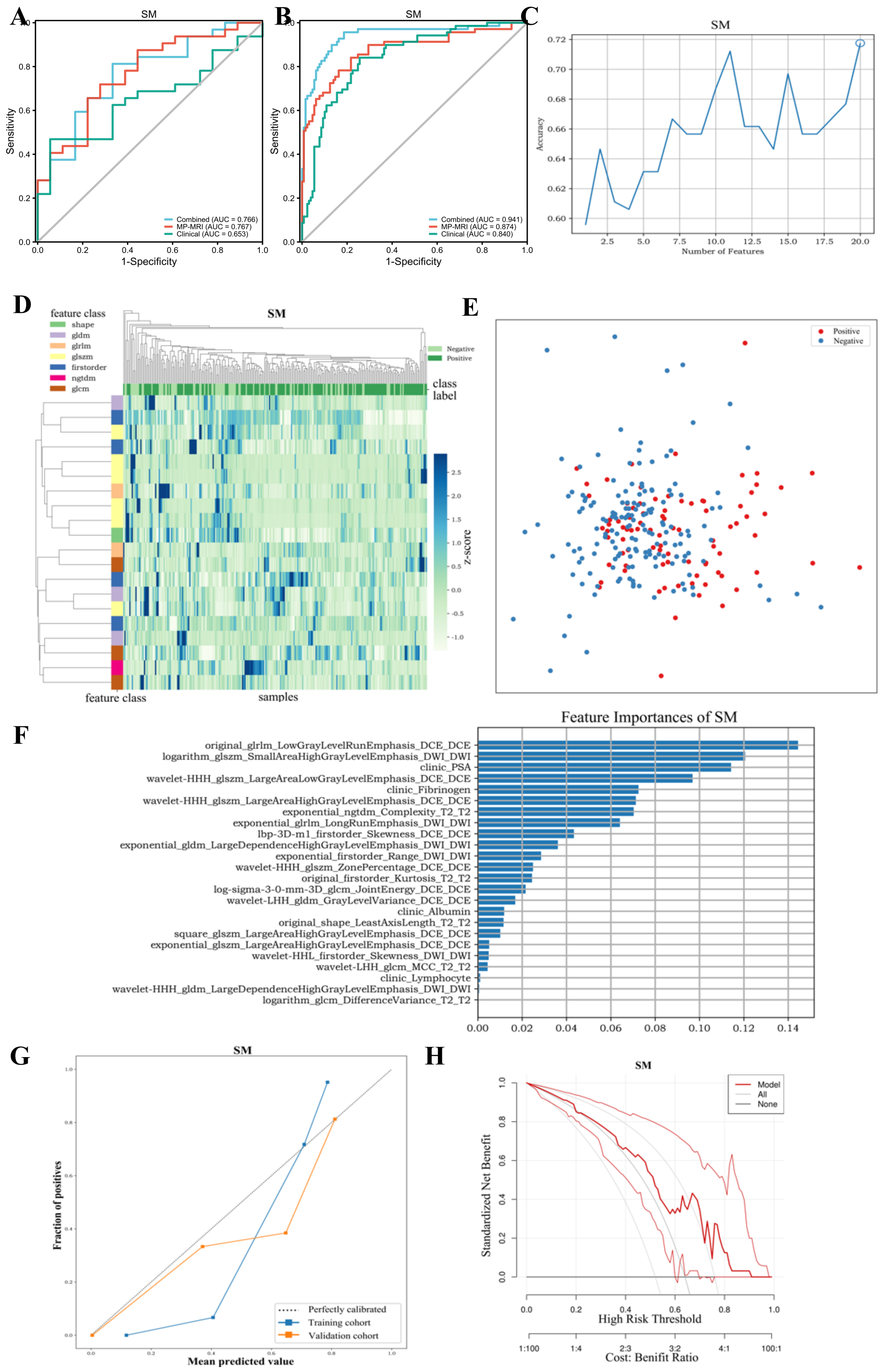

Supplementary Figure 3. The interrelationship between radiomic features.

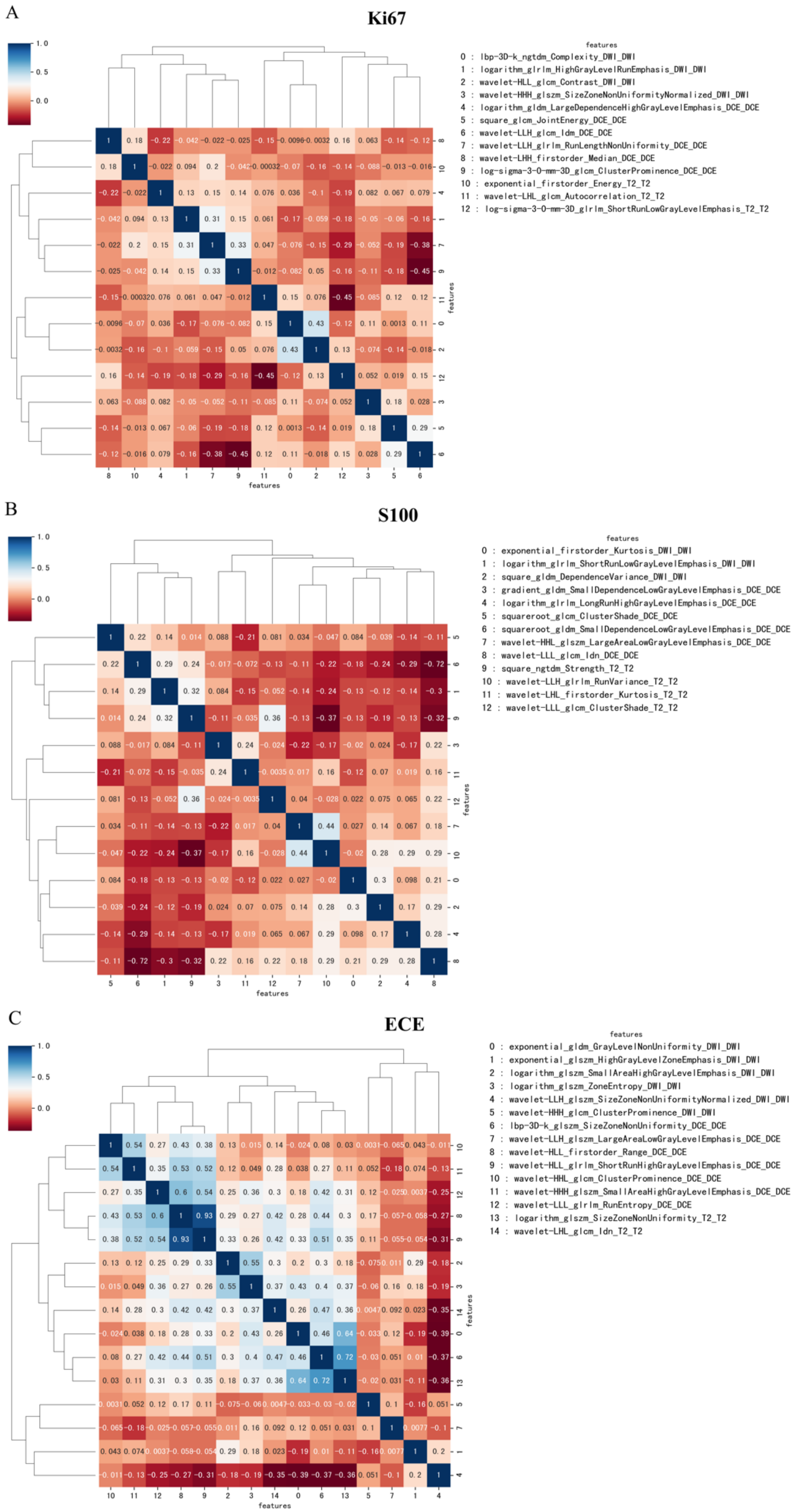

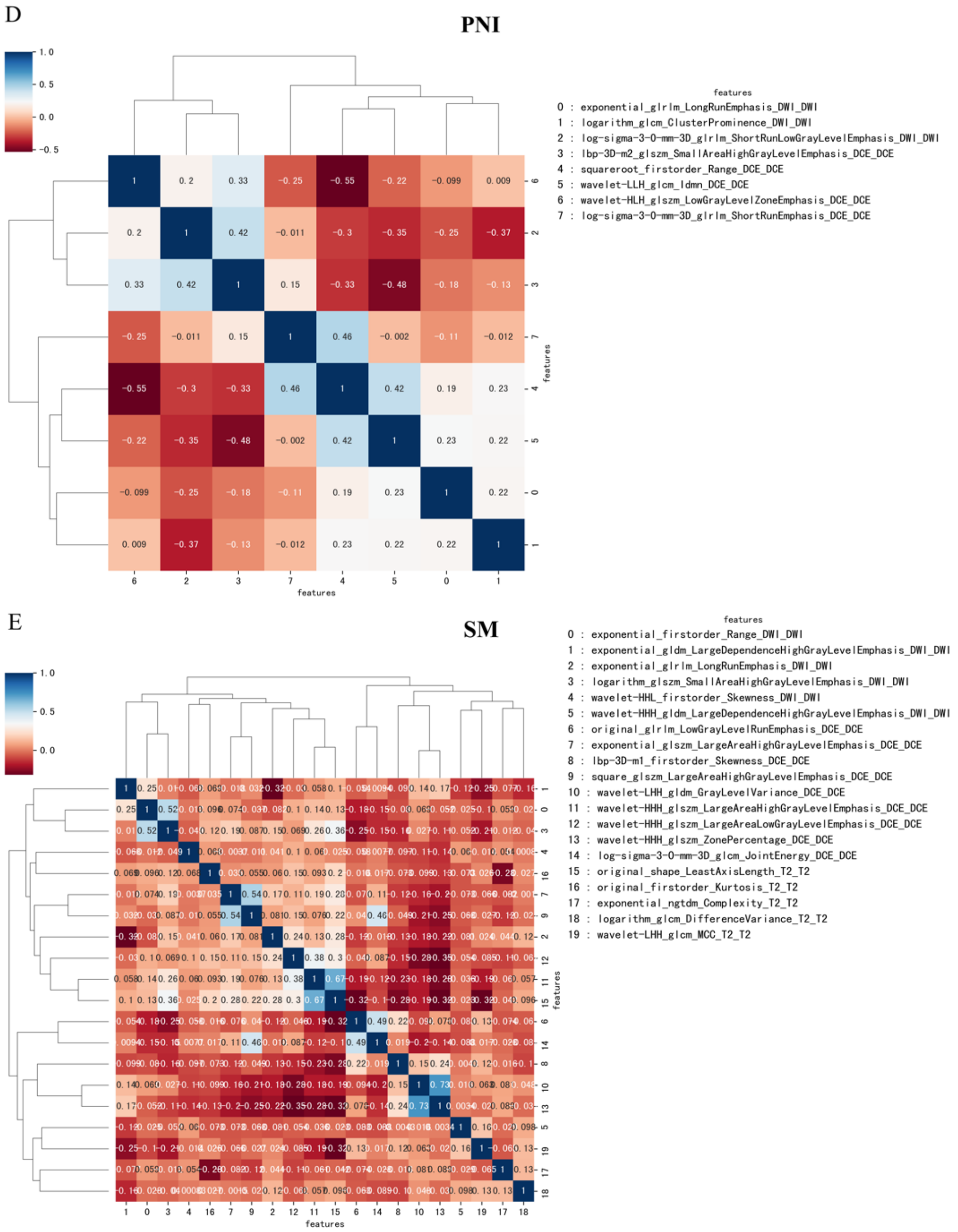

Figure S3. The interrelationship between radiomic features. The numbers on the graph represent the Pearson correlation coefficients between features, which showed there was low redundancy among selected features.
